# Additional records of *Lycodon yunnanensis* (Werner, 1922) and range extension of *Lycodon truongi* Nguyen, Duong, Wood & Grismer, 2022 (Reptilia, Squamata, Colubridae) from Vietnam

**DOI:** 10.3897/BDJ.14.e189836

**Published:** 2026-10-23

**Authors:** Anh Van Pham, Cuong The Pham, Hanh Thi Ngo, Ha L.T. Trinh, Thuy Xuan Dao, Thai Hong Pham, Nhiet Ba Pham, Chien Anh Dao, Minh Duc Le

**Affiliations:** 1 Faculty of Environmental Sciences, University of Science, Vietnam National University, Hanoi, Vietnam Faculty of Environmental Sciences, University of Science, Vietnam National University Hanoi Vietnam; 2 Institute of Biology, Vietnam Academy of Science and Technology, Hanoi, Vietnam Institute of Biology, Vietnam Academy of Science and Technology Hanoi Vietnam; 3 Graduate University of Science and Technology, Vietnam Academy of Science and Technology, Hanoi, Vietnam Graduate University of Science and Technology, Vietnam Academy of Science and Technology Hanoi Vietnam; 4 VNU-Central Institute for Natural Resources and Environmental Studies, Hanoi, Vietnam VNU-Central Institute for Natural Resources and Environmental Studies Hanoi Vietnam; 5 Department of Oceanic Science and Technology, Faculty of Hydrology, Meteorology, and Oceanography, University of Science, Vietnam National University, Hanoi, Hanoi, Vietnam Department of Oceanic Science and Technology, Faculty of Hydrology, Meteorology, and Oceanography, University of Science, Vietnam National University, Hanoi Hanoi Vietnam; 6 Chu Mom Ray National Park, Sa Thay, Vietnam Chu Mom Ray National Park Sa Thay Vietnam; 7 Faculty of Environmental Sciences, University of Science, Vietnam National University, Hanoi, Hanoi, Vietnam Faculty of Environmental Sciences, University of Science, Vietnam National University, Hanoi Hanoi Vietnam

**Keywords:** molecular phylogeny, morphology, new records, taxonomy, Truong’s wolf snake

## Abstract

**Background:**

Vietnam is home to 19 species of wolf snakes, genus *Lycodon*. Over the last five years, six new species have been discovered in the country.

**New information:**

*Lycodon yunnanensis* (Werner) is reported for the first time from Ha Tinh Province, Vietnam. Genetic analysis of the mitochondrial cytochrome *b* (cyt*b*) and cytochrome c oxidase subunit I (*COI*) fragments shows that their genetic distances are below 1.65% and 2.89%, respectively. In addition, new records of *Lycodon truongi* Nguyen, Duong, Wood & Grismer are reported from central Vietnam, based on eight specimens collected in Da Nang City and Quang Ngai Province. Although the newly-collected specimens exhibit some morphological variations from the holotype specimen, the genetic divergence between samples from the populations is estimated to be less than 2.0%, based on a fragment of cyt*b*.

## Introduction

The genus *Lycodon* Boie, 1827 is one of the most diverse genera of colubrid snakes, with 85 currently recognised species (Uetz et al. (Eds) 2026). The members of this genus have a broad distribution from eastern Iran eastwards to Japan, southwards to the Philippines as well as the Indo-Australian Archipelago (Lanza 1999, Siler et al. 2013, Neang et al. 2014, Janssen et al. 2019). Thirteen new species of the genus have been described in the last five years, namely *L. anakradaya* Nguyen, Duong, Wood & Grismer; *L. calcarophilus* Vogel, Bragin, Poyarkov & Nguyen; *L. cathaya* Wang, Qi, Lyu, Zeng & Wang; *L. chithrasekarai* Wickramasinghe, Vidanapathirana, Pushpamal & Wickramasinghe; *L. deccanensis* Ganesh, Deuti, Punith, Achyuthan, Mallik, Adhikari & Vogel; *L. duytan* Nguyen, Poyarkov & Vogel; *L. irwini* Naveen, Mirza, Choure & Chandramouli; *L. latifasciatus* Nguyen, Lee, Jiang, Ding, Chit, Poyarkov & Vogel; *L. neomaculatus* Nguyen, Lee, Pauwels, Kennedy-Gold, Poyarkov, David & Vogel; *L. poyarkovi* Nguyen & Vogel; *L. serratus* Wang, Yu, Vogel & Che; *L. truongi* Nguyen, Duong, Wood & Grismer and *L. zayuensis* Jiang, Wang, Jin & Che (Ganesh et al. 2020, Wang et al. 2020, Wickramasinghe et al. 2020, Nguyen and Vogel 2025, Nguyen et al. 2025a, Nguyen et al. 2025b, Uetz et al. (Eds) 2026). In Vietnam, to date 19 species of *Lycodon* have been documented, including *L. anakradaya*; *L. calcarophilus; L. capucinus* Boie; *L. cardamomensis* Daltry & Wüster; *L. chapaensis* (Angel & Bourret); *L. davisonii* (Blanford); *L. duytan*; *L. fasciatus* (Anderson); *L. futsingensis* (Pope); *L. laoensis* Günther; *L. meridionalis* (Bourret); *L. neomaculatus*; *L. paucifasciatus* Rendahl in Smith; *L. pictus* Janssen, Pham, Ngo, Le, Nguyen & Ziegler; *L. poyarkovi*; *L. rufozonatus* Cantor; *L. ruhstrati abditus* Vogel, David, Pauwels, Sumontha, Norval, Hendrix, Vu & Ziegler; *L. truongi* and *L. yunnanensis* (Werner) (Janssen et al. 2019, Nguyen et al. 2022, Nguyen and Vogel 2025, Uetz et al. (Eds) 2026).

*Lycodon yunnanensis* (Werner, 1922) was described from Kunming City, Yunnan Province, China and is known to occur in Yunnan (Chuxiong, Lincang, Dali, Yuxi, Mengzi cities, Jingdong, Jianshui and Jinping counties in China) and Vietnam (Vogel et al. 2024). *Lycodon truongi* was recently described from Khanh Trung Commune, Khanh Vinh District, Khanh Hoa Province, Vietnam, based on a single male holotype (Nguyen et al. 2022) and this species was subsequently reported from Binh Thuan Province (now known as Lam Dong Province) by Phan et al. (2025). During our fieldwork in central Vietnam, additional specimens of *L. yunnanensis* and *L. truongi* were collected from Da Nang City, Ha Tinh and Quang Ngai provinces. We herein record *L. yunnanensis* for the first time from Ha Tinh Province and report new provincial records of *L. truongi*.

## Materials and methods

**Sampling**

Field surveys were conducted in April 2014 and in October 2024 in Chu Mom Ray National Park (NP) (Quang Ngai Province); in April 2020, in the Elephant Habitat and Species Conservation Area (HSCA) and in December 2024, in Nam Giang Protection Forest (Da Nang City); and in April 2025, in Vu Quang National Park (Ha Tinh Province) Vietnam. Specimens were collected between 19:00 h and 23:00 h. After having been photographed in life, snakes were anaesthetised and euthanised in a closed vessel with a piece of cotton wool containing ethyl acetate, fixed in 85% ethanol for ten hours and later transferred to 75% ethanol for permanent storage. Tissue samples were preserved separately in 70% ethanol before fixation. Voucher specimens were deposited in the collections of the University of Science (HUS), Vietnam National University, Hanoi (VNU) and the Institute of Biology (IB), Vietnam Academy of Science and Technology, Hanoi, Vietnam.

**Molecular data and phylogenetic analyses**

DNA was extracted using the DNeasy Blood and Tissue Kit (Qiagen, Germany), following the manufacturer’s instructions. Two fragments of the mitochondrial genes, the cytochrome *b* (cyt*b*) and cytochrome c oxidase subunit I (COI), were amplified using the primer pair L14910 (5’–GACCTGTGATMTGAAAACCAYCGTTGT-3’) and H16064 (5’– CTTTGGTTTACAAGAACAATGCTTTA-3’ (Burbrink et al. 2000)) and RepCOIF (5’-TNTTMTCAACNAACCACAAAGA-3’) and RepCOIR (5’-ACTTCTGGRTGKCCAAARAATCA-3’) (Nagy et al. 2012). Extracted DNA was amplified using HotStarTaq Mastermix (Qiagen, Germany) with 21 µl volume consisting of 10 µl of Mastermix, 5 µl of water, 2 µl of each primer at 10 pmol/ml and 2 µl of DNA. PCR conditions were 95°C for 15 minutes to activate the Taq, with 40 cycles of 95°C for 30 s, 45°C for 45 s, 72°C for 60 s and a final extension at 72°C for six minutes. PCR products were visualised using gel electrophoresis through a 2% low melting-point agarose gel stained with ethidium bromide. Successful amplifications were purified to eliminate PCR components using the GeneJET^TM^ PCR Purification Kit (ThermoFisher Scientific, Lithuania). Purified PCR products were sent to FirstBase (Malaysia) for sequencing.

The obtained sequences were aligned in ClustalX 2.1 (Thompson et al. 1997) using the default settings. Data were then analysed using Maximum Parsimony (MP) as implemented in PAUP*4.0b10 (Swofford 2001), Maximum Likelihood (ML) in IQ-TREE v.3.0.1 (Wong et al. 2025) and Bayesian Inference (BI) in MrBayes v.3.2.7 (Ronquist et al. 2012). Settings for MP and BI analyses followed Le et al. (2006), except that the number of generations in the Bayesian analysis was increased to 1×10^7^. For the Maximum Likelihood (ML) analysis, we used IQ-TREE v.3.0.1 (Wong et al. 2025) with a single model and 10,000 ultrafast bootstrap replications and the best fit model for nucleotide evolution was set to TIM2+F+I+G4, as determined by ModelFinder implemented in IQ-TREE v.3.0.1 (Kalyaanamoorthy et al. 2017). For BI, the optimal model for nucleotide evolution was set to GTR+I+G by jModelTest v.2.1.10 (Posada and Crandall 1998). Four Markov chains, one cold and three heated (utilising default heating values), were sampled every 1,000 generations. Log-likelihood scores of sample points were plotted against generation time to detect stationarity of the Markov chains. The cut-off point for the burn-in function was set to 25% of the total number of trees generated in the BI analysis. Two independent analyses were run simultaneously. Nodal support was evaluated using ultrafast bootstrap replication (UFB) as estimated in IQ-TREE v.3.0.1, bootstrap replication (BP) as calculated in PAUP and posterior probability (PP) as determined in MrBayes v.3.2.7. BP ≥ 70% and UFB and PP ≥ 95% are regarded as strong support for a clade (Hillis and Bull 1993, Ronquist et al. 2012, Hoang et al. 2018). Uncorrected pairwise distances (p) were calculated in PAUP.

**Morphological examination**

Measurements were taken with a digital caliper (Electronic Digital Caliper) to the nearest 0.1 mm. The following morphological characteristics were recorded (after Janssen et al. (2019), Luu et al. (2019)): SVL Snout-vent length (from tip of snout to vent); TaL tail length; TaL/TL ratio of tail length/total length; TL total length = SVL + TaL; DSR dorsal scale rows number at one head length posterior to the head – number of dorsal scale rows at mid-body – number of dorsal scale rows at one head length anterior to the vent; SL supralabials (counted on upper lips); SL/orbit number of supralabials entering orbit; IL infralabials (counted on lower lips); Lor loreals; Lor/eye loreal scale touching the eye (yes or no); PreOc pre-oculars; PostOc postoculars; Atem number of anterior temporals; Ptem number of posterior temporals; BodySc scalation of the body (keeled or smooth); PreVen number of preventral scales; Ven number of ventral scales; SubC number of subcaudal scales; Prec precloacal (or cloacal) plate (single or divided); Teeth max number of maxillary teeth/alveoli. Scalation: Scale counts were taken following Vogel et al. (2009). Ventral scales (Ven) were counted according to Dowling (1951). Bilateral scale counts were given as left/right.

## Taxon treatments

### Lycodon yunnanensis

Werner, 1922

8C770798-8958-5831-8468-EAEF87ADC27E

#### Materials

- **Type status:** Other material. **Occurrence:** catalogNumber: HUS.2025 R. 15; individualCount: 1; sex: male; lifeStage: adult; occurrenceID: 90FCE2C0-BAEC-5631-A364-2B20A4414AB8; **Taxon:** scientificNameID: *Lycodon yunnanensis* ; scientificName: *Lycodon yunnanensis*; class: Reptilia; order: Squamata; family: Colubridae; genus: *Lycodon*; specificEpithet: *yunnanensis* ; scientificNameAuthorship:  Vogel & David, 2010; **Location:** country: Vietnam; countryCode: VN; stateProvince: Ha Tinh; county: Vu Quang; locality: Vu Quang NP; verbatimElevation: 1980; verbatimLatitude: 14°25'562"N; verbatimLongitude: 107°43'254"E; verbatimCoordinateSystem: WGS84; **Event:** eventDate: 24Apri 2025; eventRemarks: collected by Pham VA; **Record Level:** language: en; basisOfRecord: Preserved Specimen

#### Description

**Figure 1.**
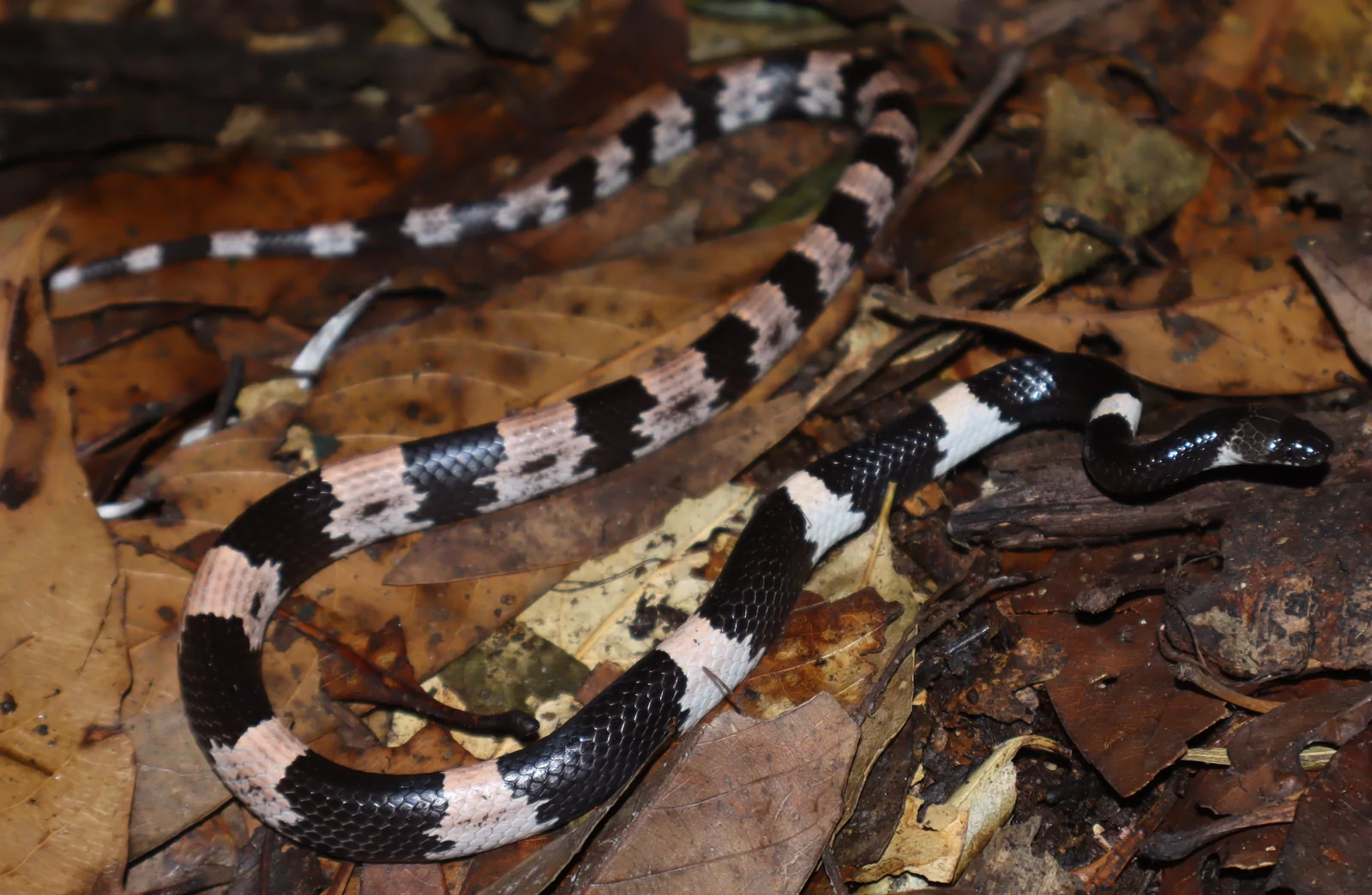
*Lycodon yunnanensis* ( HUS.2025.15 ) in life, adult male. Photos: A.V. Pham.

**Figure 2.**
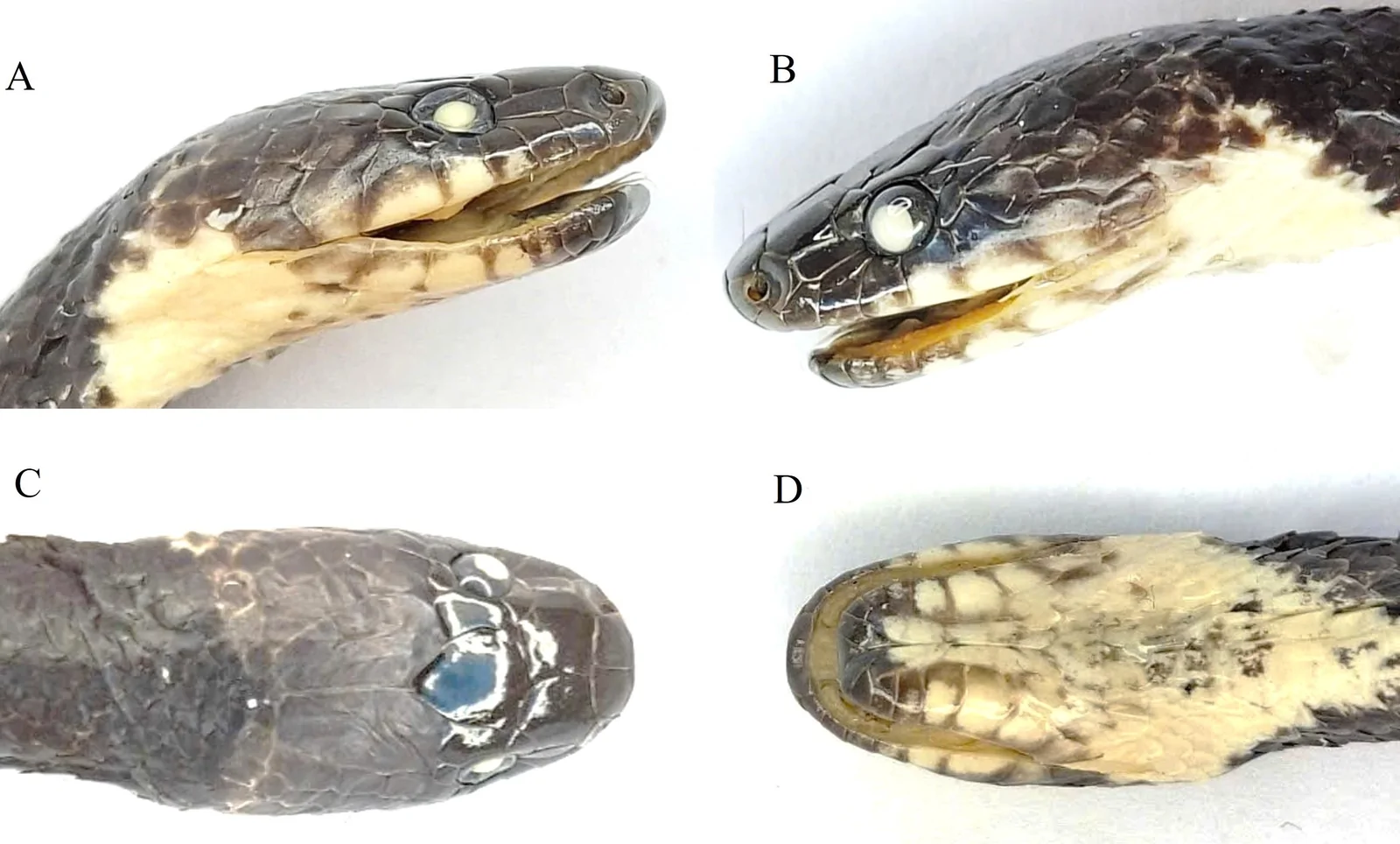
*Lycodon yunnanensis* (HUS.2025.15) in preservative: Head, lateral view (**A, B**), dorsal view (**C**); and ventral view (**D**). Photos: A. V. Pham.

Morphological characters of the specimen from Ha Tinh Province were consistent with those reported in the descriptions of Vogel et al. (2024) (Figs 1, 2): SVL 380.5 mm, TaL/TL ratio 0.20; head elongate, moderately distinct from neck, rather flattened, longer than wide, narrow anteriorly; maxillary teeth 16; nostril lateral, located in the middle of the nasal; eye large, pupils vertically elliptic; rostral triangular, broader than high, hardly visible from above; internasals two, curved, slightly wider than long, in contact with rostral anteriorly, nasal and prefrontal; two prefrontals, large, subrectangular, slightly shorter than frontal; frontal single, enlarged, subpentagonal frontal; parietals longer than wide, in contact with each other medially, with upper anterior and posterior temporals, paraparietal laterally and three nuchal scales posteriorly; paraparietals longer than wide; loreal 1/1, elongate, entering orbit; supralabials 8/8, first and second in contact with nasal, third to fifth entering orbit, seventh supralabial largest; infralabials 9/9, first pair in broad contact with each other, first to fifth in contact with anterior pair of chin shields; anterior and posterior pairs of chin shields elongate, of the same size and shape, second pair not meeting in mid-line 1/5; pre-ocular 1/1; postoculars 2/3, bordering anterior temporals; anterior temporals 2/2, posterior temporals 3/3; preventral 1, ventrals 203, from behind neck region distinctly notched laterally; subcaudals 79, paired; precloacal plate single; DSR 17–17–15, the vertebral scales not enlarged (Table 1).

**Colouration in life**. Head uniformly blackish-brown dorsally and laterally; dorsal surface of body and tail dark blackish-brown, with 20 greyish-pinkish-cream dorsal bands and nine light bands on tail; first body band starting at ventral scale 17 and slightly extended laterally to a width of 3–6 scales; throat cream-coloured; ventral surface mostly cream with black cross-bands, more regular and a few small dark spots posteriorly; ventral surface of tail black grey with light cross-bands (Fig. 1).

#### Distribution

The species was known to occur in Yunnan, China and north-eastern Vietnam (Vogel et al. 2024). The new record of this species is located in Ha Tinh Province, Vietnam, the southernmost occurrence documented of the species being approximately 470 km from the nearest location as stated in Vogel et al. (2024).

#### Ecology

Specimen was found on the ground in leaf litter of evergreen forest at 21:10 h. The surrounding habitat was evergreen forest with large, medium and small hardwoods mixed with shrubs.

### Lycodon truongi

Nguyen, Duong, Wood & Grismer, 2022

AC85DB6D-9B00-5628-8B29-96C2ADD9B084

#### Materials

- **Type status:** Other material. **Occurrence:** catalogNumber: IB R.6388; individualCount: 1; sex: female; lifeStage: adult; occurrenceID: 2C3D0046-0F5C-50F5-9B69-49F3F2275A9F; **Taxon:** scientificNameID: *Lycodon truongi*; scientificName: *Lycodon truongi*; class: Reptilia; order: Squamata; family: Colubridae; genus: *Lycodon*; specificEpithet: *truongi*; scientificNameAuthorship: Nguyen, Duong, Wood & Grismer, 2022; **Location:** country: Vietnam; countryCode: VN; stateProvince: Quang Ngai; locality: Chu Mom Ray NP; verbatimElevation: 675; verbatimLatitude: 14°25'562"N; verbatimLongitude: 107°43'254"E; verbatimCoordinateSystem: WGS84; **Event:** eventDate: 2 April, 2014; eventRemarks: collected by Pham TC; **Record Level:** language: en; basisOfRecord: Preserved Specimen
- **Type status:** Other material. **Occurrence:** catalogNumber: IB R.6389; individualCount: 1; sex: male; lifeStage: adult; occurrenceID: 06955724-BA59-5044-BBB3-F79FB3BCEAF5; **Taxon:** scientificNameID: *Lycodon truongi*; scientificName: *Lycodon truongi*; class: Reptilia; order: Squamata; family: Colubridae; genus: *Lycodon*; specificEpithet: *truongi*; scientificNameAuthorship: Nguyen, Duong, Wood & Grismer, 2022 ; **Location:** country: Vietnam; countryCode: VN; stateProvince: Quang Ngai; locality: Chu Mom Ray NP; verbatimElevation: 754; verbatimLatitude: 14°26'283"N; verbatimLongitude: 107°43'290"E; verbatimCoordinateSystem: WGS84; **Event:** eventDate: Apri 1, 2014; eventRemarks: collected by Pham TC; **Record Level:** language: en; basisOfRecord: Preserved Specimen
- **Type status:** Other material. **Occurrence:** catalogNumber: IB R.6390; individualCount: 1; sex: male; lifeStage: adult; occurrenceID: B2EA4120-2BFD-5983-A37C-14E30BC90900; **Taxon:** scientificNameID: *Lycodon truongi*; scientificName: *Lycodon truongi*; class: Reptilia; order: Squamata; family: Colubridae; genus: *Lycodon*; specificEpithet: *truongi*; scientificNameAuthorship: Nguyen, Duong, Wood & Grismer, 2022 ; **Location:** country: Vietnam; countryCode: VN; stateProvince: Da Nang ; locality: Elephant HSCA; verbatimElevation: 105; verbatimLatitude: 15°34'466"N; verbatimLongitude: 107°55'980"E; verbatimCoordinateSystem: WGS84; **Event:** eventDate: Apri 27, 2020; eventRemarks: collected by Pham TC and Phan QT; **Record Level:** language: en; basisOfRecord: Preserved Specimen
- **Type status:** Other material. **Occurrence:** catalogNumber: IB R.6391; individualCount: 1; sex: female; lifeStage: adult; occurrenceID: 8AF4C4CF-00AD-52CE-A7DE-3A35463D9FAC; **Taxon:** scientificNameID: *Lycodon truongi*; scientificName: *Lycodon truongi*; class: Reptilia; order: Squamata; family: Colubridae; genus: *Lycodon*; specificEpithet: *truongi*; scientificNameAuthorship: Nguyen, Duong, Wood & Grismer, 2022 ; **Location:** country: Vietnam; countryCode: VN; stateProvince: Da Nang ; locality: Nam Giang Protection Forest; verbatimElevation: 509; verbatimLatitude: 15°71'595"N; verbatimLongitude: 107°60'365"E; verbatimCoordinateSystem: WGS84; **Event:** eventDate: December 4, 2024; eventRemarks: collected by Pham TC and Phan QT; **Record Level:** language: en; basisOfRecord: Preserved Specimen
- **Type status:** Other material. **Occurrence:** catalogNumber: IB R.6392; individualCount: 1; sex: male; lifeStage: adult; occurrenceID: 48B0C579-BA7F-5630-9D1F-977E4DA28C1A; **Taxon:** scientificNameID: *Lycodon truongi*; scientificName: *Lycodon truongi*; class: Reptilia; order: Squamata; family: Colubridae; genus: *Lycodon*; specificEpithet: *truongi*; scientificNameAuthorship: Nguyen, Duong, Wood & Grismer, 2022 ; **Location:** country: Vietnam; countryCode: VN; stateProvince: Da Nang ; locality: Nam Giang Protection Forest; verbatimElevation: 509; verbatimLatitude: 15°71'595"N; verbatimLongitude: 107°60'365"E; verbatimCoordinateSystem: WGS84; **Event:** eventDate: December, 2024; eventRemarks: collected by Pham TC and Phan QT; **Record Level:** language: en; basisOfRecord: Preserved Specimen
- **Type status:** Other material. **Occurrence:** catalogNumber: HUS 2025 R.01; individualCount: 1; sex: male; lifeStage: adult; occurrenceID: A70CE825-4A40-5AE9-8510-370C781A19E3; **Taxon:** scientificNameID: *Lycodon truongi*; scientificName: *Lycodon truongi*; class: Reptilia; order: Squamata; family: Colubridae; genus: *Lycodon*; specificEpithet: *truongi*; scientificNameAuthorship: Nguyen, Duong, Wood & Grismer, 2022 ; **Location:** country: Vietnam; countryCode: VN; stateProvince: Quang Ngai; locality: Chu Mom Ray NP; verbatimElevation: 660; verbatimLatitude: 14°26'003"N; verbatimLongitude: 107°43'136"E; verbatimCoordinateSystem: WGS84; **Event:** eventDate: October 1, 2024; eventRemarks: collected by Le DM and Pham VA; **Record Level:** language: en; basisOfRecord: Preserved Specimen
- **Type status:** Other material. **Occurrence:** catalogNumber: HUS 2025 R.02; individualCount: 1; sex: male; lifeStage: adult; occurrenceID: 8A8B0B3C-24D4-51EB-857F-F4CA02A04EDC; **Taxon:** scientificNameID: *Lycodon truongi*; scientificName: *Lycodon truongi*; class: Reptilia; order: Squamata; family: Colubridae; genus: *Lycodon*; specificEpithet: *truongi*; scientificNameAuthorship: Nguyen, Duong, Wood & Grismer, 2022 ; **Location:** country: Vietnam; countryCode: VN; stateProvince: Quang Ngai; locality: Chu Mom Ray NP; verbatimElevation: 660; verbatimLatitude: 14°26'003"N; verbatimLongitude: 107°43'136"E; verbatimCoordinateSystem: WGS84; **Event:** eventDate: October, 2024; eventRemarks: collected by Le DM and Pham VA; **Record Level:** language: en; basisOfRecord: Preserved Specimen
- **Type status:** Other material. **Occurrence:** catalogNumber: HUS 2025 R.03; individualCount: 1; sex: male; lifeStage: adult; occurrenceID: 85D55A11-F713-5D5E-80E2-BA91E8E28FB5; **Taxon:** scientificNameID: *Lycodon truongi*; scientificName: *Lycodon truongi*; class: Reptilia; order: Squamata; family: Colubridae; genus: *Lycodon*; specificEpithet: *truongi*; scientificNameAuthorship: Nguyen, Duong, Wood & Grismer, 2022 ; **Location:** country: Vietnam; countryCode: VN; stateProvince: Quang Ngai; locality: Chu Mom Ray NP; verbatimElevation: 460; verbatimLatitude: 14°31'313"N; verbatimLongitude: 107°36'401"E; verbatimCoordinateSystem: WGS84; **Event:** eventDate: October, 2024; eventRemarks: collected by Le DM and Pham VA; **Record Level:** language: en; basisOfRecord: Preserved Specimen

#### Description

**Figure 3.**
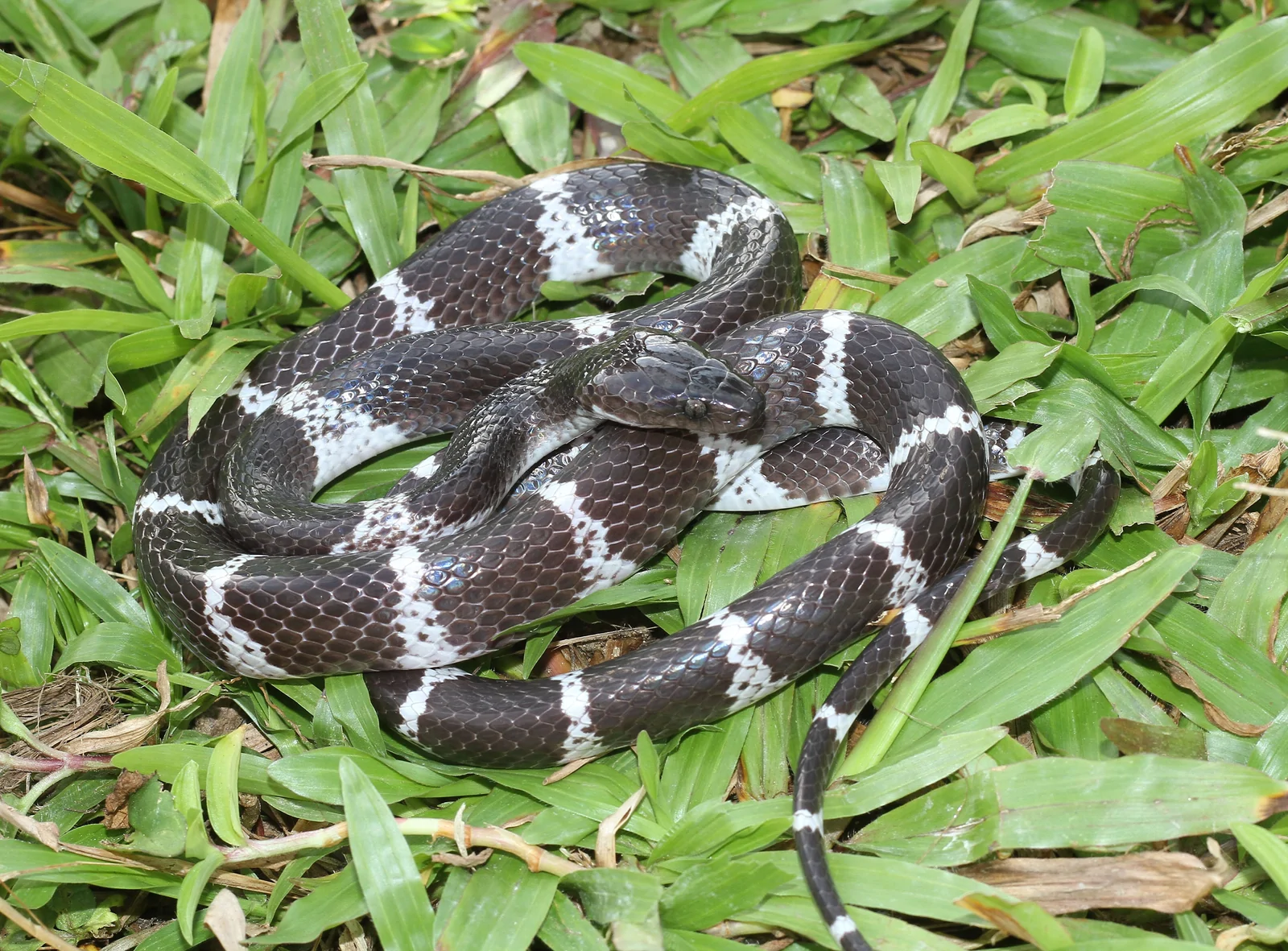
*Lycodon truongi* (IB R.6391) in life, adult female. Photos: C.T. Pham.

**Figure 4.**
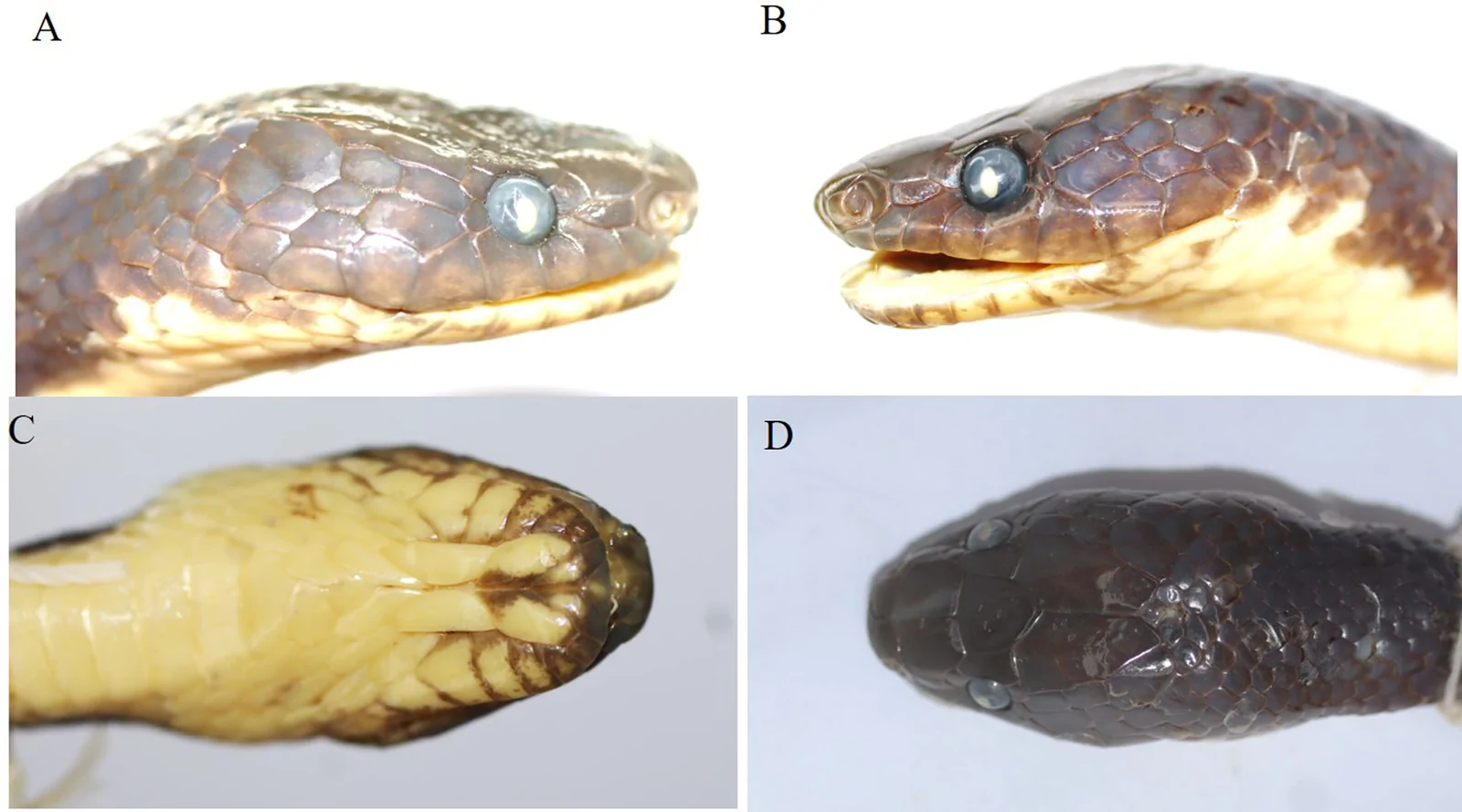
*Lycodon truongi* (IB R.6388) in preservative: Head, **A, B** lateral view; **C** ventral view; **D** dorsal view. Photos: A. V. Pham.

**Figure 5.**
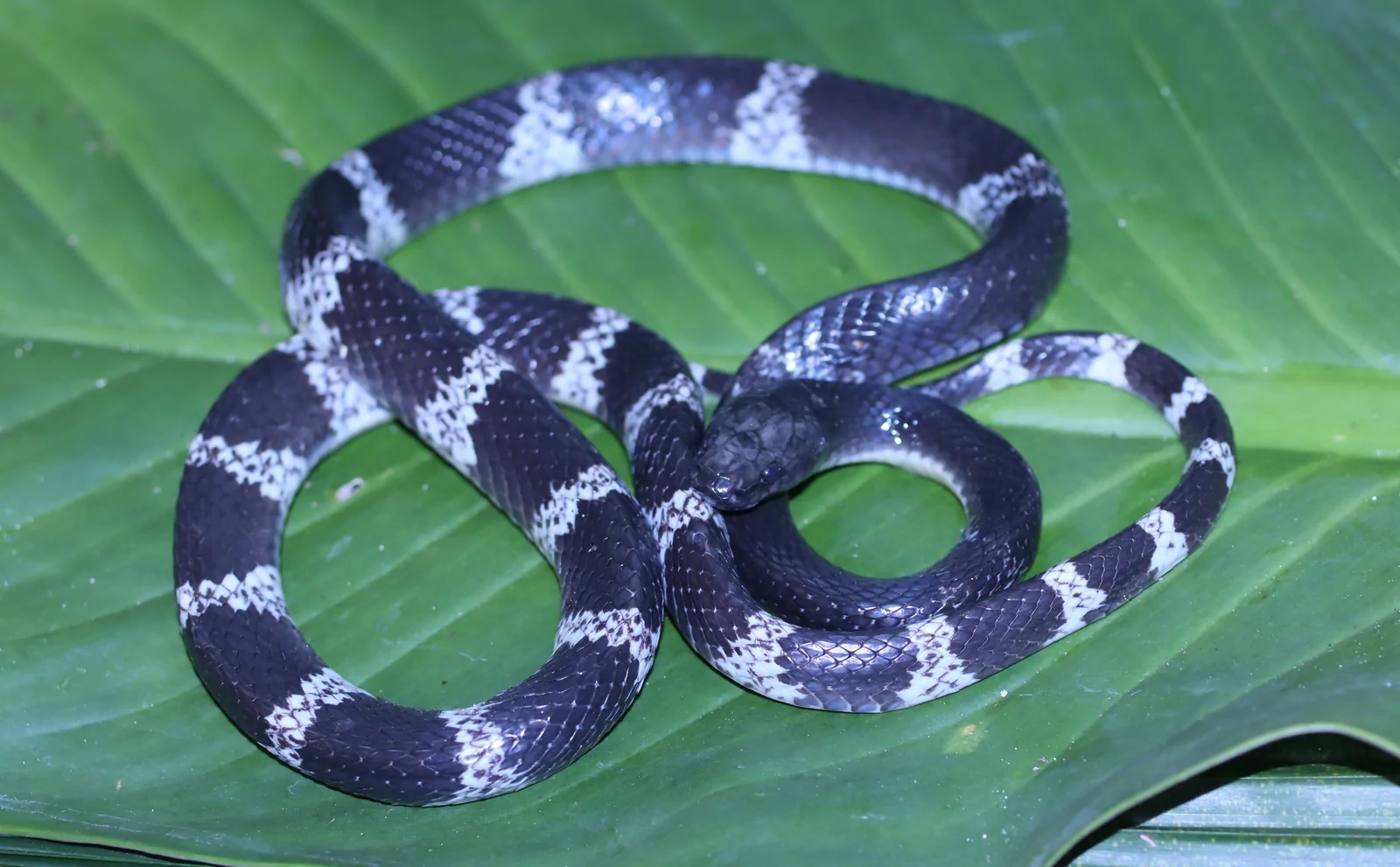
*Lycodon truongi* (HUS.2025.02) in life, adult male. Photos: A.V. Pham.

Morphological characters of the specimens from Da Nang City and Quang Ngai Province were consistent with those reported in the descriptions of Nguyen et al. (2022), Phan et al. (2025): Maximal SVL 881.3 mm in males and 626.7 mm in female; TaL/TL ratio 0.208–0.244; maxillary teeth 19–26; dorsal scales in 17–17–15 rows, 4–8 weakly keeled vertebral scale rows from mid-body to vent; supralabials 8/8; infralabials 10/10; one elongated loreal on each side, in contact or not with the eye; anterior and posterior pairs of chin shields elongate, of the same size and shape, second pair not meeting in mid-line 1/3; pre-ocular 1/1; postoculars 2/2; anterior temporals 2/2; posterior temporals 3/3, upper ones thinner than lower ones; precloacal plate single; ventral scales 197–210; subcaudals 81–91; dorsal surface of body with 19–25 narrowed greyish-cream bands; dorsal surface of tail with 13–15 cream bands; ventral surface cream with a few small dark spots posteriorly; ventral surface of tail dark grey with irregular blotches corresponding to light crossbands (Table 1, Figs 3, 4, 5).

#### Distribution

*Lycodon truongi* is currently known from Da Nang City and Quang Ngai, Khanh Hoa and Binh Thuan (now known as Lam Dong) provinces, Vietnam.

#### Ecology

Specimens were found on the ground in leaf litter of evergreen forest at night between 19:00 h and 23:00 h. The surrounding habitat was evergreen forest with large, medium and small hardwoods mixed with shrubs. Air temperatures at the sites ranged from 20.0–30.0°C and relative humidity was 70–90%.

## Analysis

**Phylogenetic analyses**

**Figure 6.**
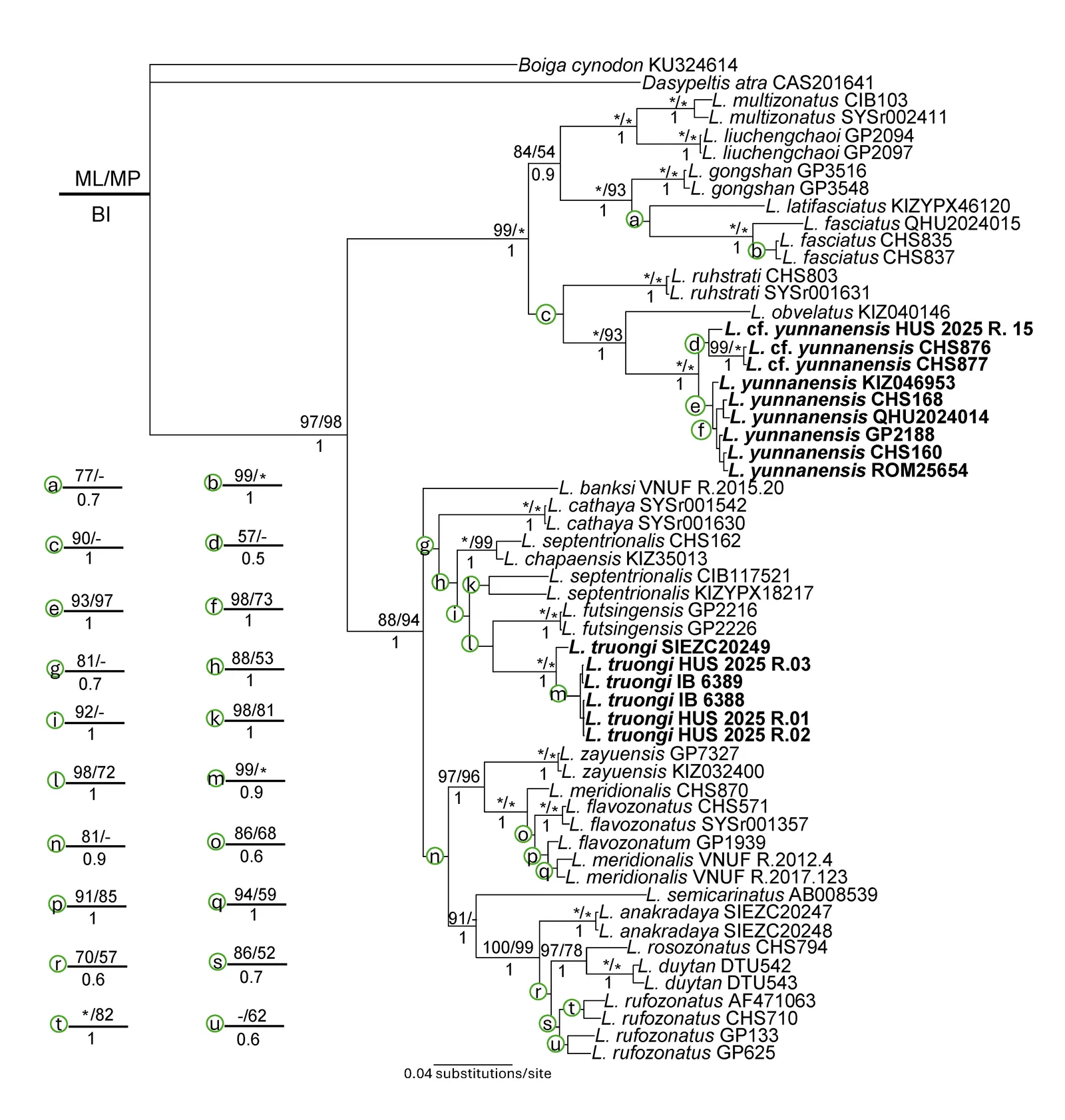
Phylogram, based on the Bayesian analysis. Numbers above and below branches are MP bootstrap/ML ultrafast bootstrap values and single-model Bayesian posterior probabilities (> 50%), respectively. Dash indicates unsupported node. Letters and numbers after species names are GenBank accession numbers.

The final matrix consisted of 1,707 bp aligned characters (COI: 657 and cyt*b*: 1050), of which 1,044 were constant, 526 parsimony informative and 137 variable characters parsimony-uninformative. The MP analysis produced a single most parsimonious tree (tree length = 2,051, consistency index = 0.43, retention index = 0.74). Our tree topologies were very similar to those recovered by Guo et al. (2013), Luu et al. (2018), Nguyen et al. (2022). The sequences of the specimens from Chu Mom Ray NP clustered with that of *L. truongi*, with strong support in both analyses (PP = 1, UFB = 100, BP = 100). The new sequences had an uncorrected p-distance of less than 2.0% compared with that of the holotype of *Lycodon truongi*, based on a fragment of the cyt*b* gene (Fig. 6, Suppl. material 1). Additionally, sequences of the specimen from Ha Tinh Province embedded within *L. yunnanensis* with strong support in all analyses (PP = 1, UFB = 100, BP = 100). The genetic divergence between sequences of *L. yunnanensis* from Ha Tinh and China was estimated equal or below 2.06% and 2.89%, based on cyt*b* and COI fragments, respectively.

## Discussion

Taxonomic revision of *Lycodon* has been a major focus of recent studies in colubrid snakes. Over the last five years, 13 new species of the genus have been described (Uetz et al. (Eds) 2026). Amongst them, six taxa, *L. anakradaya*, *L. calcarophilus*, *L. duytan, L. pictus*, *L. poyarkovi*, and *L. truongi*, have recently been discovered in Vietnam (Janssen et al. 2019, Nguyen et al. 2022, Nguyen et al. 2025a, Nguyen et al. 2025b, Nguyen and Vogel 2025).

*Lycodon yunnanensis* was described by Vogel et al. (2024) from Yunnan (Chuxiong, Lincang, Dali, Yuxi, Mengzi cities, Jingdong, Jianshui and Jinping counties, China). The newly-documented locality in Vietnam is approximately 350 km from the type locality in China. We found *L. yunnanensis* on the ground near the streams in evergreen forest. Other sympatric amphibian and reptile species were also encountered, including *Leptobrachella eos* (Ohler, Wollenberg, Grosjean, Hendrix, Vences, Ziegler & Dubois), *Leptobrachium chapaense* (Bourret), *Limnonectes bannaensis* Ye, Fei, Xie & Jiang, *Xenophrys lancangica* Lyu, Wang & Wang, *Odorrana* sp. and *Acanthosaura lepidogaster* (Cuvier).

Nguyen et al. (2022) described *Lycodon truongi* from Khanh Hoa Province, Vietnam and considered the new species a member of the *Lycodon rufozonatus* species complex. However, the description was based on a single specimen and since then only one study has been carried out to determine the species distribution and morphological variations. Our results show that the species has a broader range and additional surveys can even expand its currently known distribution. We found *L. truongi* on the ground near streams in evergreen forest. Other sympatric amphibian and reptile species were also observed, including *Leptobrachella firthi* (Rowley, Hoang, Dau, Le & Cao), *Leptobrachium pullus* (Smith), *Limnonectes bannaensis* (Ye, Fei, Xie & Jiang), *Ophryophryne hansi* (Ohler), *Xenophrys lancangica* (Lyu, Wang & Wang), *Odorrana chloronota* (Günther), *Scincella* sp. and *Trimeresurus albolabris* (Gray). This species has not been reported in disturbed areas and, thus, is strongly associated with evergreen tropical forests. Habitat throughout much of this species' range continues to decline in quality and extent due to agricultural activities and tourism (Sodhi et al. 2009).

We recommend listing *Lycodon truongi* as Least Concern (LC), based on the IUCN Red List Categories and Criteria (IUCN 2026), because it is widely distributed and relatively abundant at the surveyed sites. Further research is needed to clarify the population status and extent of occurrence of this species and to determine threats resulting from habitat loss and degradation and poaching.

## Supplementary Material

XML Treatment for Lycodon yunnanensis

XML Treatment for Lycodon truongi

B5A64B85-473C-5008-94B9-AD35211FECBF 10.3897/BDJ.14.e189836.suppl1 Supplementary material 1 Genetic distance between species of the genus *Lycodon* included in this study Data type Excel table Brief description Uncorrected (“p”) distance matrix showing percentage pairwise genetic divergence (*COI* and Cyt*b*) between *Lycodon* species. File: oo_1651981.xlsx https://binary.pensoft.net/file/1651981 Anh Van Pham, Cuong The Pham, Hanh Thi Ngo, Ha Le Thi Trinh, Thuy Xuan Dao, Thai Hong Pham, Nhiet Ba Pham, Chien Anh Dao, Minh Duc Le

## Figures and Tables

**Table 1.** Morphological characteristics of *Lycodon yunnaneisis* and *L. truongi* from southern Vietnam.

	*Lycodon yunnanensis*	*Lycodon truongi*							
	HUS 2025 R. 15	HUS 2025 R.01	HUS 2025 R.02	HUS 2025 R.03	IB R.6389	IB R.6388	IB R.6390	IB R.6391	IB R.6392
Sex	M	M	M	M	M	F	M	F	M
TL	475.3	1043.8	827.4	931.1	899.5	761	653.6	763.2	1086.2
SVL	380.5	881.3	625.6	767.5	712.6	599.5	542.2	626.7	847.1
TaL	94.8	162.5^+^	201.8	163.6^+^	186.9	161.5	111.4^+^	136.5^+^	239.1
TaL/TL	0.19	-	0.244	-	0.208	0.212	-	-	0.220
SL	8/8	8/8	8/8	8/8	8/8	8/8	8/8	8/8	8/8
SL/ orbit	3-5/3-5	3-5/3-5	3-5/3-5	3-5/3-5	3-5/3-5	3-5/3-5	3-5/3-5	3-5/3-5	3-5/3-5
Teeth	16/16	26/26	26/26	24/24	25/25	25/25	19/19	23/23	25/25
IL	8/8	10/10	10/10	10/10	10/10	10/10	10/10	10/10	10/10
PreOc	1/1	1/1	1/1	1/1	1/1	1/1	1/1	1/1	1/1
PostOc	3/3	2/2	2/2	2/2	2/2	2/2	2/2	2/2	2/2
Lor	1/1	1/1	1/1	1/1	1/1	1/1	1/1	1/1	1/1
InN/Lor	Y	Y	Y	Y	Y	y	Y	Y	Y
Lor/eye	Y	No	No	Y	Y	No	Y	No	Y
Atem	2/2	2/2	2/2	2/2	2/2	2/2	2/2	2/2	2/2
Ptem	3/3	3/3	3/3	3/3	3/3	3/3	3/3	3/3	3/3
DSR	19-17-15	17-17-15	17-17-15	17-17-15	17-17-15	17-17-15	17-17-15	17-17-15	17-17-15
PreVen	2	2	2	2	2	2	2	2	2
Ven	204	206	201	203	200	200	198	202	197
PreC		entire	entire	entire	entire	entire	entire	entire	entire
SubC	81	52^+^	82	56^+^	81	81	88	60^+^	89
BodySc (keeled medial rows)	8	6	4	6	6	6	8	4	6
Adult head pattern	uniform black	uniform black	uniform black	uniform black	uniform black	uniform-black	uneven black	uniform-black	uniform black
Dorsal ground colour	pinkish-orange	black	black	black	black	black	black	black	black
Ventrolateral body pattern	banded	banded	banded	banded	banded	banded	banded	banded	banded
Body bands narrow or wide	thick	thin	thin	thin	thin	thin	thick	thin	thin
Light bands on body	20	22	25	22	22	22	25	23	22
Light bands on tail	9	8+	14	10+	13	13	15	9+	14
